# The Effect of Breathing Laterality on Hip Roll Kinematics in Submaximal Front Crawl Swimming

**DOI:** 10.3390/s22062324

**Published:** 2022-03-17

**Authors:** John M. Barden, Mike V. Barber

**Affiliations:** Faculty of Kinesiology and Health Studies, University of Regina, Regina, SK S4S 0A2, Canada; barberathletic@gmail.com

**Keywords:** front crawl, hip roll kinematics, accelerometry, snorkel

## Abstract

The purpose of this study was to determine the effect of breathing laterality on hip roll kinematics in submaximal front crawl swimming. Eighteen elite competitive swimmers performed three 100 m front crawl trials at a consistent sub-maximal speed (70% of seasonal best time) in a 25 m pool. Each trial was performed with one of three different breathing conditions: (1) unilateral breathing (preferred side), (2) bilateral breathing (alternating left/right-side every 3 strokes) and (3) simulated non-breathing using a swim snorkel. A waist-mounted triaxial accelerometer was used to determine continuous hip roll angle throughout the trial, from which peak hip roll angles (Ө) and average angular velocities (ω) were calculated. Two-way repeated measures ANOVAs were used to identify significant main effects for laterality (preferred vs. non-preferred breathing sides) and condition (unilateral, bilateral and snorkel breathing) for both Ө and ω. Peak hip roll to the preferred side was significantly greater (*p* < 0.001) in the unilateral condition, while ω to the non-preferred side was significantly greater in the unilateral (*p* < 0.01) and bilateral (*p* < 0.04) conditions. Significant same-side differences were also found between the different breathing conditions. The results demonstrate that breathing laterality affects hip roll kinematics at submaximal speeds, and that unilateral and snorkel breathing are associated with the least and most symmetric hip roll kinematics, respectively. The findings show that a snorkel effectively balances and controls bilateral hip rotation at submaximal speeds that are consistent with training, which may help to minimize and/or correct roll asymmetries that are the result of unilateral breathing.

## 1. Introduction

Front crawl requires the coordination of asynchronous arm and leg movements for swimmers to move through the water. Unlike breaststroke and butterfly, which require synchronous and bilaterally symmetric movements, the arms and legs in front crawl and backstroke move in lateral opposition to each other in a manner analogous to the gait cycle. To create propulsion, one arm applies force to the water while the other arm is being repositioned to begin the next stroke. These alternating cycles of propulsion and recovery are accompanied by rotation of the swimmer’s body about the longitudinal axis, typically known as body roll (or trunk roll), which can be measured at the shoulder and/or hip joints [1,2].

Body roll is an integral part of a swimmer’s technique in that it serves to coordinate and balance the propulsion and recovery phases on both sides of the body, and helps to increase reach at the end of the catch phase to maximize stroke length [3]. Biomechanical studies of body roll, while limited in number, show that the quantity of roll is influenced by three main factors: (1) anatomical location (shoulders or hips), (2) swimming speed and (3) breathing. Several studies have demonstrated that shoulder rotation is typically greater than hip rotation, which occurs because the trunk twists about its long axis creating relative motion (torsion) between the upper and lower trunk segments [1,4,5,6]. Other studies have shown that an inverse relationship exists between body roll and swimming speed, such that body roll decreases with increased speed [1,2,5,7,8] and to a greater extent at the hips than at the shoulders [4,9]. Finally, body roll facilitates the action of breathing [3], which produces an increased roll to the breathing side. Given that not all strokes involve breathing, several studies have controlled for the effects of breathing by analyzing only non-breathing stroke cycles [1,10], while others have demonstrated that swimmers roll their shoulders and hips significantly more to the breathing side than the non-breathing side [7,11,12,13].

To swim front crawl, competitive swimmers typically adopt one of two different breathing strategies: (1) unilateral breathing, in which a breath is taken to the same side once every two strokes (i.e., once every stroke cycle), or (2) bilateral breathing, in which a breath is taken once every three strokes, so that breathing is equally distributed between right and left sides. Unilateral breathing is common and inherently asymmetric [13], as most swimmers have a preferred breathing side that is consistent with the side used when they first learned how to swim. Given the relationship between breathing and body roll, unilateral breathing appears to be a significant factor in the development of body roll asymmetry, in which a swimmer rolls more to one side (the breathing side) than the other. Elite swim coaches recognize the importance of a balanced body roll to optimize stroke length and increase efficiency, and typically emphasize bilateral breathing and/or the use of a snorkel to prevent stroke length asymmetries. Body roll has been shown to have a significant effect on the mediolateral hand path [14,15], and as such asymmetric roll patterns have the potential to affect hand position and the resulting repetitive stress placed on the shoulder. Several researchers have suggested that insufficient body roll is associated with the development of swimmer’s shoulder [5,16,17,18,19,20,21], and recently, Vila Dieguez and Barden [9] found that swimmers with unilateral shoulder pain rolled their hips (and not their shoulders) less to the non-preferred breathing side than swimmers without shoulder pain, suggesting that hip roll asymmetry may be related to the etiology, or incidence of, shoulder impingement.

Consequently, further research is needed to understand the relationship between breathing laterality and body roll, and to our knowledge the effect of different breathing patterns, including the effect of using a swim snorkel (to simulate non-breathing conditions), on the laterality of hip roll kinematics has not been investigated. Further, given that both the magnitude and speed of rotation are important factors in the timing and coordination of the stroke cycle, it is important to investigate both aspects of hip roll kinematics, and we are unaware of any research that has investigated the effects of breathing on hip roll angular velocity. Therefore, the purpose of this study was to determine the effect of different breathing patterns on hip roll kinematics in front crawl swimming at a consistent, submaximal speed. The primary objective was to determine the extent to which hip roll angular displacement and velocity differed between preferred and non-preferred breathing sides for unilateral, bilateral and non-breathing (i.e., snorkel) conditions. It was hypothesized that unilateral breathing would be associated with greater hip roll kinematics to the breathing (preferred) side than to the non-breathing side. It was also hypothesized that the bilateral breathing and snorkel conditions would be associated with less hip roll asymmetry, such that there would be no bilateral differences in hip roll kinematics.

## 2. Materials and Methods

### 2.1. Participants

Based on peak hip roll angles from previous studies [1,22], an *a priori* power analysis revealed that a sample of eighteen participants would provide sufficient power for the statistical analyses. Consequently, eighteen elite level competitive swimmers (11 males, 7 females; see Table 1 for anthropometric data) were recruited from the local university and community swim programs. Inclusion criteria for all participants were: (1) that they had been actively training for a minimum of three years and (2) had recently achieved (in the previous 12 months) a provincial “A” qualifying standard in a front crawl event. Ethics approval was obtained from the local university ethics review board and informed written consent was obtained from each participant prior to the beginning of the study.

### 2.2. Apparatus and Procedure

**Figure 1:**
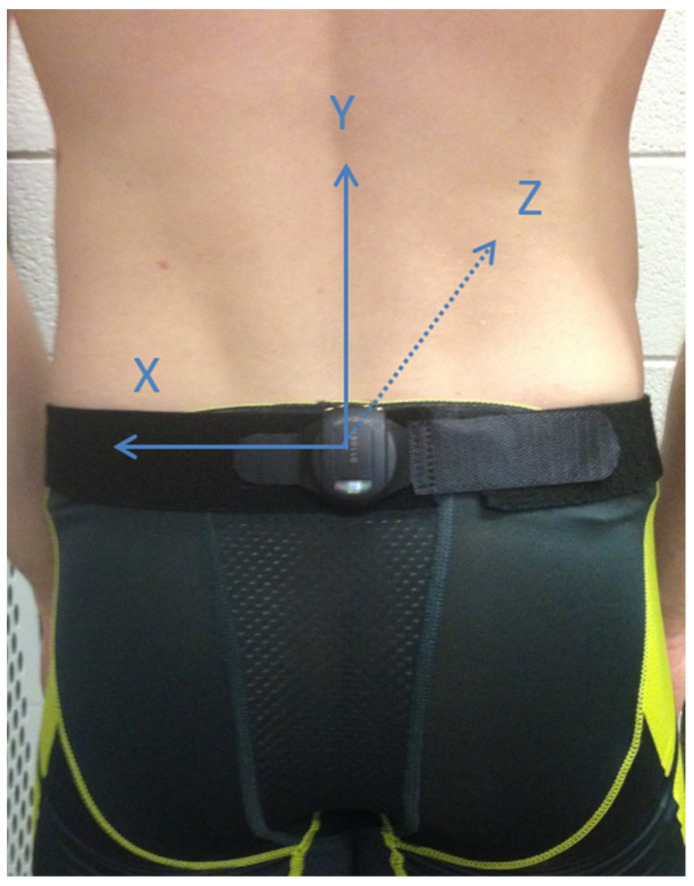
Image showing sensor orientation, placement and alignment.

Hip roll was quantified in the manner described by Bächlin & Tröster [23] and implemented by Barden & Barber [24] and Vila Dieguez & Barden [9], in which a single, waterproof tri-axial accelerometer (Activinsights, Cambridgeshire, UK) was attached to an elastic belt that was securely fastened around the waist at the level of the L5 vertebra (see Figure 1). The accelerometer sampled at 100 Hz and was oriented so that the Y-axis was in line with the spine, the X-axis was perpendicular to the Y-axis (mediolateral with respect to the swimmer), and the Z-axis was oriented along the anteroposterior axis of the swimmer’s body (i.e., vertical with respect to the horizontal position of the swimmer in the water).

A calibration procedure was implemented in which participants laid on the pool deck in the prone position for approximately 10 s. This position was used to ensure that zero degrees of roll (the neutral position in the frontal plane) about the longitudinal axis of the swimmer was parallel to the pool deck. Any bias in the sensor orientation about the roll axis (typically less than 1°) was averaged and removed to correct the calculated angle to the zero degree reference position. A gravity reference calibration was also employed to ensure that the position of the sensor did not deviate throughout the trial. With the sensor oriented horizontally (i.e., 0° about the pitch axis) in the prone position with no inertial acceleration on the mediolateral (X) or anteroposterior (Z) axes, the acceleration due to gravity will align with the Z-axis, which will have X and Z components as the sensor rotates about the Y-axis. The instantaneous X and Z-axis accelerations throughout each trial were checked to ensure that they were equal to 1 g. The mean gravitational (XZ) acceleration for all participants (18) across all lengths (4) of all three trials was 0.99 g with a standard deviation of 0.02 g.

Participants performed a typical 15–20 min warm-up prior to the start of the test session. The session consisted of 3 × 100 m front crawl trials in a 25 m indoor pool, at a sub-maximal velocity equivalent to 70% of the participant’s season best 100 m front crawl time (i.e., 30% was added to their seasonal best time). Because swimming at higher speeds reduces hip roll, this velocity was selected to represent a comfortable intensity that was consistent with a swimmer’s typical hip roll pattern during training. Each of the three trials required the participants to swim with a different breathing pattern: (1) unilateral breathing (breathing to the preferred side every two strokes), (2) bilateral breathing (breathing to each side once every 3 strokes) and (3) a snorkel (simulated non-breathing) trial, in which participants swam with a specially designed swim snorkel (FINIS, Tracy, CA, USA) so the head did not have to turn to breathe. All participants were familiar with snorkel swimming and the trial order was randomized for each participant. Each 100 m trial was timed with a stopwatch to ensure that a consistent velocity was maintained between trials. All trials began from a push start in the pool and participants were allowed a recovery period of 3 min between trials to mitigate any effects of fatigue.

### 2.3. Data and Statistical Analysis

**Figure 2:**
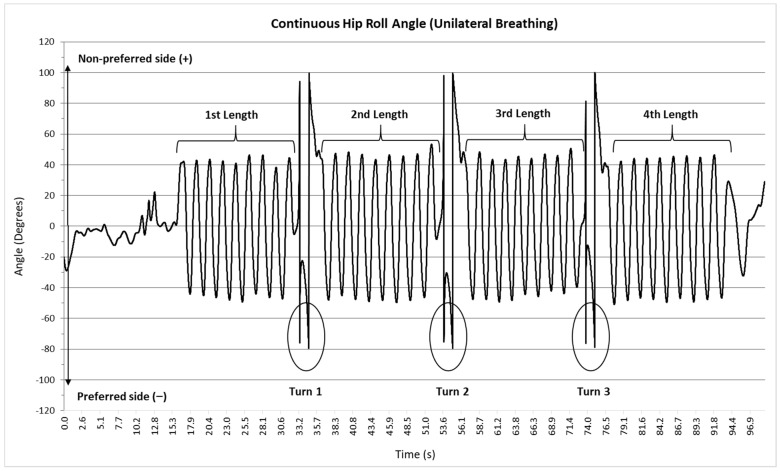
Representative continuous hip roll angle for each length of a 100 m unilateral (preferred side) breathing trial for a single participant.

**Figure 3:**
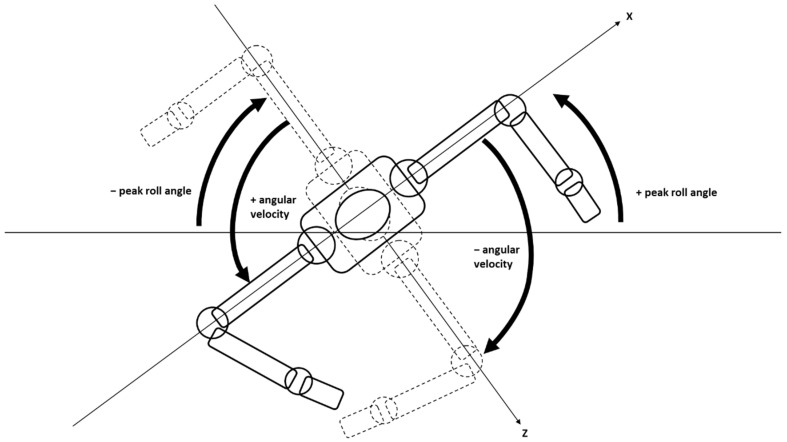
Diagram showing the determination of peak clockwise (−) and counter-clockwise (+) hip rotation and average clockwise (i.e., from peak + hip roll angle to peak − hip roll angle) and counter-clockwise angular velocity.

Raw acceleration data were processed using custom Matlab scripts (MathWorks, Natick, MA, USA) that filtered the data with a 4th order Butterworth digital filter with a lowpass cutoff frequency of 1 Hz. This cutoff frequency was selected because the mean fundamental frequency for hip roll for all participants across all trials was 0.54 Hz. The continuous hip roll angle for each trial was calculated using the filtered acceleration data and the tangential equation method for the X and Z axes [9,23]. A representative trial for unilateral breathing is shown in Figure 2. Positive (counter-clockwise) and negative (clockwise) peak hip roll angles for each stroke were determined and averaged for each length of the trial. These angles were matched to the participants’ self-reported preferred and non-preferred breathing sides. Positive and negative angular velocities (ω) were determined for each stroke using the first central difference method, which were used to determine the average angular velocity for that stroke (i.e., from peak hip roll angle on one side to peak hip roll angle on the other side). The average angular velocities for each stroke were averaged for each length of the trial and matched to the participants preferred and non-preferred breathing sides (see Figure 3).

To analyze the data, a series of repeated-measures ANOVAs were used to determine the effect of breathing condition on hip roll kinematics to the preferred and non-preferred breathing sides. The first ANOVA (one-way) used trial condition as the within-subject variable to ensure that there were no significant differences between trials with respect to time (which served as a proxy for speed). Next, length was used as the within-subject variable (L1, L2, L3 and L4) to ensure that there were no between-length differences in hip roll kinematics for the three 100 m trials. This analysis allowed the four lengths of each trial to be averaged to obtain mean peak hip roll angles and average angular velocities for the preferred and non-preferred breathing sides for each condition. Two-way repeated measures ANOVAs were then implemented to determine differences for Ө and ω for breathing condition (unilateral, bilateral and snorkel) and breathing side laterality (preferred vs. non-preferred). Bonferroni-adjusted comparisons were used to further identify any significant interaction effects. The level of statistical significance was set to *p* < 0.05.

## 3. Results

No significant differences were found between trials with respect to time, demonstrating that all three trials were completed at an equivalent speed. Similarly, no significant differences were found between lengths, indicating that hip roll kinematics were consistent for all four lengths of each trial and could be combined for further analysis.

The results of the statistical analyses for peak hip roll angle and average angular velocity are summarized in Table 2.

Significant main effects were found for both breathing condition and side for Ө and ω, as were significant interaction effects (see Table 2). For Ө, the pairwise comparisons for side (preferred vs. non-preferred) revealed that peak hip roll angle was significantly greater to the preferred side in the unilateral breathing condition (*p* < 0.001, mean difference = 9.61, 95% CI = [6.1, 13.2]), but not for the other two conditions (see Table 2). Pairwise comparisons for condition (unilateral vs. bilateral vs. snorkel) revealed that peak hip roll angle on the preferred side was significantly less for the snorkel condition compared to both the unilateral (*p* < 0.001, mean difference = 6.95, 95% CI = [2.7, 11.2]) and bilateral breathing conditions (*p* < 0.001, mean difference = 4.38, 95% CI = [2.3, 6.4]). 

For ω, post-hoc comparisons for side (preferred vs. non-preferred) revealed that average angular velocity to the non-preferred side was significantly greater for both the unilateral (*p* < 0.01, mean difference = 6.17, 95% CI = [1.6, 10.7]) and bilateral (*p* < 0.04, mean difference = 3.29, 95% CI = [0.2, 6.4]) conditions. For condition (unilateral vs. bilateral vs. snorkel), the comparisons showed that preferred side angular velocity was significantly greater for the bilateral condition compared to the unilateral (*p* < 0.001, mean difference = 3.86, 95% CI = [1.9, 5.9]) and snorkel conditions (*p* < 0.001, mean difference = 5.49, 95% CI = [3.0, 8.0]), while non-preferred angular velocity for the snorkel condition was significantly less than the unilateral (*p* < 0.01, mean difference = −6.56, 95% CI = [−11.1, −2.1]) and bilateral conditions (*p* < 0.001, mean difference = −7.54, 95% CI = [−10.5, −4.6]).

## 4. Discussion

The purpose of this study was to determine the effect of breathing laterality on hip roll kinematics during front crawl swimming at a consistent submaximal speed. The hypothesis that unilateral breathing would be associated with greater hip roll kinematics to the preferred breathing side was partially supported, in that peak hip roll angles to the preferred side were significantly greater than to the non-preferred side. However, the average angular velocity toward the preferred side (i.e., from peak hip roll angle on the non-breathing side to peak hip roll angle on the breathing side) was significantly less, not greater, than the velocity in the opposite direction (i.e., from the breathing side to the non-breathing side). It was also hypothesized that the bilateral and snorkel conditions would be associated with more symmetric (i.e., balanced) hip roll kinematics, with no significant differences between preferred and non-preferred sides. This hypothesis was supported for peak hip roll and for average angular velocity in the snorkel condition, but not for velocity in the bilateral condition, as similar to the unilateral condition, the average velocity to the non-preferred side was significantly greater than to the preferred side.

The angular displacement results for unilateral breathing demonstrate that the mean difference in peak hip roll angle between breathing and non-breathing sides was 9.6°, which is consistent with the findings of Kippenhan and Hay [25] and Payton et al. [12], who found differences between breathing and non-breathing upper body roll angles of 8 and 9°, respectively. Peak body roll angles for the breathing (66°) and non-breathing (57°) sides in the Payton et al. [12] study were slightly greater than in the current study, which likely occurred because upper body roll angle (i.e., shoulder roll) was measured as opposed to hip roll. These results are also consistent with the findings of Vila Dieguez and Barden [9], who found differences in hip roll angle between breathing and non-breathing sides at slow, medium and fast speeds of 8°, 8° and 9°, respectively.

For the bilateral breathing and snorkel conditions, no significant differences were found between preferred and non-preferred sides, even though peak hip roll angles to the preferred side continued to be greater in both conditions. While the mean difference between sides did not reach the level of statistical significance (4.7° and 2.3° for bilateral and snorkel breathing, respectively), the results suggest the possibility of an underlying preference for rolling to the preferred side, regardless of whether breathing occurs to that side or not. Given that the difference in roll angle between sides was lowest for the snorkel condition, the results suggest that breathing may exaggerate the presence of an underlying asymmetry. The findings also show that the use of a snorkel produced significant reductions in preferred side hip roll relative to the unilateral (−7.0°) and bilateral conditions (−4.4°), while hip roll to the opposite (non-preferred) side did not change. This demonstrates that the snorkel successfully balanced hip roll kinematics, and did so more effectively than bilateral breathing (in which preferred side hip roll did not decrease significantly relative to the unilateral condition).

For hip roll angular velocity, the findings were contrary to what was expected as stated in the hypothesis. A significant difference between the preferred (breathing) and non-preferred sides was found in the unilateral breathing condition, but it was the angular velocity towards the side with the least amount of roll (the non-preferred side) that was greater, not the reverse as predicted by the hypothesis. This significant difference was also found for the bilateral condition. The results demonstrate that hip rotation velocity was greater when the participants rolled away from the side with the greatest angular displacement, not towards it. To our knowledge, this is the first study to report hip roll angular velocity in front crawl swimming, so a comparison of the results to other studies is not possible. However, Payton et al. [12] noted that the increased roll angle associated with breathing requires the torso to rotate through a greater range of motion to return to the neutral position. Consequently, a slight increase in the speed of rotation following a breathing side roll might help to maintain the timing and coordination of the stroke, in particular the coupling of body rotation with the pull phase of the contralateral arm (i.e., the negatively directed roll that follows a breath to the left side will coincide with the right pull phase, and vice versa). Given the oppositional pattern of the arm stroke cycle, the increased angular velocity to the non-breathing side could be a compensatory action, in response to the increased hip rotation on the breathing side, which serves to maintain the timing of the stroke. While additional data is needed to support this hypothesis, it’s consistent with what would be expected if stroke efficiency is to be maintained in the presence of a breathing-related asymmetry.

It’s also important to note that this kinematic pattern (i.e., greater preferred side hip roll and greater non-preferred side angular velocity) persisted in the bilateral condition when the breathing was equally distributed to both sides, which supports the idea of a persistent roll asymmetry to the preferred side. Consequently, in addition to breathing laterality, the current results suggest differences in bilateral motor patterns, which are likely the result of greater familiarity and repetition of breathing to the preferred side. In relation to this point, peak hip roll angles to the non-preferred side changed very little across all three conditions (even when breathing in the bilateral condition), and were essentially the same between the unilateral and snorkel conditions (given that both values represented non-breathing hip roll), which is consistent with the idea of a persistent, preferred-side breathing asymmetry. Similarly, angular velocity to the non-preferred side was significantly reduced in the snorkel condition relative to the unilateral and bilateral conditions, which was presumably a result of the reduction in the preferred side roll angle due to the lack of breathing when using the snorkel.

Finally, it is important to note that this study had several limitations. While a formal validation of the sensor-based method to determine hip/body roll angles has not been published, it has been used previously [9] based on internal test trial comparisons with 2D videography that suggest an approximate error of 1–3 degrees. Given that the current results are consistent with equivalent hip roll angles found in the literature, it is expected that any errors were systematic errors that did not affect the statistical comparisons or final results. For future studies that employ a sensor-based method to investigate body roll kinematics in front crawl swimming, it is recommended to use an inertial measurement unit (IMU), as it contains a rate gyroscope that can be used to provide an additional data source (angular velocity) for angular kinematic verification. IMU sensors were not used in the current study as they were not available at the time.

## 5. Conclusions

This study showed that the amount of hip rotation was greater when breathing unilaterally to the preferred side, whereas the angular velocity was greater when rolling to the non-preferred side. In addition, both bilateral and snorkel breathing successfully reduced unilateral roll asymmetry, but snorkel breathing was the only condition that eliminated significant bilateral differences in hip roll angular velocity. The results demonstrate that the most effective way to balance hip roll kinematics and mitigate roll asymmetry is to use a swim snorkel.

## Figures and Tables

**Table 1:** Participant characteristics and 100 m trial data. Values are mean (±1) standard deviation.

	Males (n = 11)	Females (n = 7)
Age (years)	19.4 (2.0)	19.1 (1.0)
Height (cm)	180.8 (7.8)	171.4 (3.1)
Weight (kg)	79.7 (8.2)	67.3 (7.2)
Body mass index (kg/m^2^)	24.4 (8.0)	22.9 (5.2)
100m SB time (s)	55.2 (1.9)	59.0 (2.4)
Mean Trial Time (s)	73.7 (3.0)	76.5 (3.0)
Percentage of SB ^1^	74.9 (0.02)	77.2 (0.02)
Average velocity (m∙s^−1^)	1.36 (0.06)	1.31 (0.02)

^1^ SB = seasonal best.

**Table 2:** Summary of Results.

				ANOVA Results		
Variable	Condition	Side	Mean (SD)	Effect	*p* Value	η^2^p
Ө (°)	Unilateral **	Preferred *	54.2 (8.8)	Side	0.03	0.24
	Unilateral	Non-preferred *	44.6 (8.0)	Condition	0.001	0.56
	Bilateral †	Preferred	51.6 (9.9)	Interaction	0.01	0.25
	Bilateral	Non-preferred	46.9 (10.2)			
	Snorkel **†	Preferred	47.2 (9.2)			
	Snorkel	Non-preferred	44.9 (10.1)			
ω (°/s)	Unilateral **	Preferred *	100.3 (14.3)	Side	0.03	0.24
	Unilateral §	Non-preferred *	106.5 (17.7)	Condition	0.001	0.55
	Bilateral **†	Preferred *	104.2 (14.5)	Interaction	0.001	0.33
	Bilateral ††	Non-preferred *	107.5 (16.7)			
	Snorkel †	Preferred	98.7 (14.7)			
	Snorkel §††	Non-preferred	99.9 (15.8)			

* = significant difference between sides; **, †, § and †† = significant differences between conditions.

## Data Availability

Informed consent obtained from the subjects involved in this study does not cover public sharing of the data. The data may be available upon request from the corresponding author and pending approvals.
